# Tissue-Specific Responses of IGF-1/Insulin and mTOR Signaling in Calorie Restricted Rats

**DOI:** 10.1371/journal.pone.0038835

**Published:** 2012-06-06

**Authors:** Naveen Sharma, Carlos M. Castorena, Gregory D. Cartee

**Affiliations:** 1 Muscle Biology Laboratory, School of Kinesiology, University of Michigan, Ann Arbor, Michigan, United States of America; 2 Department of Molecular and Integrative Physiology, University of Michigan, Ann Arbor, Michigan, United States of America; 3 Institute of Gerontology, University of Michigan, Ann Arbor, Michigan, United States of America; University of Padova, Italy

## Abstract

Moderate calorie restriction (CR) (∼60% of ad libitum, AL, intake) has been associated with numerous favorable physiological outcomes in many species, and the insulin/IGF-1 and mTOR signaling pathways have each been proposed as potential mediators for many of CR's bioeffects. However, few studies have assessed the widely held idea that CR induces the down-regulation of the insulin/IGF-1 and/or mTOR pathways in multiple tissues. Accordingly, we analyzed the phosphorylation status of 11 key signaling proteins from the insulin/IGF-1 (IR^Tyr1162/1163^, IGF-1R^Tyr1135/1136^, IRS-1^Ser312^, PTEN^Ser380^, Akt^Ser473^, GSK3α^Ser21^, GSK3β^Ser9^) and mTOR (TSC2^Ser939^, mTOR^Ser2448^, P70S6K^Thr412^, RPS6^Ser235/236^) pathways in 11 diverse tissues [liver, kidney, lung, aorta, two brain regions (cortex and cerebellum), and two slow-twitch and three fast-twitch skeletal muscles] from 9-month-old male AL and CR Fischer 344 x Brown Norway rats. The rats were studied under two conditions: with endogenous insulin levels (i.e., AL>CR) and with insulin infused during a hyperinsulinemic-euglycemic clamp so that plasma insulin concentrations were matched between the two diet groups. The most striking and consistent effect of CR was greater pAkt in 3 of the 5 skeletal muscles of CR vs. AL rats. There were no significant CR effects on the mTOR signaling pathway and no evidence that CR caused a general attenuation of mTOR signaling across the tissues studied. Rather than supporting the premise of a global downregulation of insulin/IGF-1 and/or mTOR signaling in many tissues, the current results revealed clear tissue-specific CR effects for the insulin signaling pathway without CR effects on the mTOR signaling pathway.

## Introduction

Calorie restriction (CR) without malnutrition (∼60–75% of ad libitum, AL, intake) has striking biological effects on a wide range of important outcomes (including improved health, delayed aging and increased longevity) in many species [1], [2], [3]. The underlying mechanisms for these CR-associated benefits remain unclear, but several lines of evidence have implicated the evolutionarily conserved insulin/IGF-1 signaling pathway as a potential mediator for significant aspects of the CR phenotype. Interest in the insulin/IGF-1 signaling pathway is in part attributable to the recognition that marked reductions in circulating insulin and moderate decrements in IGF-1 levels are hallmarks of CR [1], [4], [5]. Furthermore, mutations in insulin/IGF-1 signaling genes of diverse species (yeast, nematodes, drosophila, mice and rats) have been reported to be accompanied by many outcomes that are similar to CR (i.e., delayed aging and increased maximal lifespan) [1], [5], [6]. It has been suggested that “by maintaining plasma insulin at a markedly low level throughout life, CR is in effect decreasing insulin signaling” [4]. Remarkably, however, the idea that insulin signaling is reduced by CR in multiple tissues in vivo, an elemental premise for many of the models of the mechanisms that underlie CR's bioeffects, has been the subject of a limited amount of careful experimentation. The previous studies on CR and the insulin signaling pathway have rarely included the assessment of more than one tissue, and the tissue studied has usually been a single skeletal muscle without regard to muscle fiber type differences. This narrow perspective does not adequately address the idea that CR may have significant effects on many tissues.

The target of rapamycin (TOR; mTOR in mammals) pathway is also an evolutionarily conserved signaling pathway that is both responsive to nutrient intake implicated as an important modulator of many age-related diseases and maximal lifespan [7], [8], [9], [10], [11], [12], [13]. Genetic and pharmacologic interventions that down-regulate the TOR pathway in primitive organisms and rodents have been reported to result in improved health, delayed age-related dysfunction and lifespan extension. These outcomes are reminiscent of the effects of either CR or selected genetic deficits in the insulin/IGF-1 pathway [8], [10]. The TOR pathway has been described as “the prime candidate” to explain the benefits of CR on lifespan [8]. Compared to the insulin/IGF-1 pathway, even less is known about the influence of CR on mTOR signaling in mammalian tissues.

The insulin/IGF-1 and mTOR signaling pathways have each been widely invoked as likely mediators for many of CR's bioeffects [1], [4], [5], [6], [8], [14], [15], [16], [17], [18]. Because there is extensive cross-talk between the TOR and insulin/IGF-1 pathways [8], [15], [19], [20], it would be especially valuable to perform simultaneous assessment of the effects of CR on both pathways in multiple tissues. By analyzing the abundance and phosphorylation status of 11 key signaling proteins from the insulin/IGF-1 (IR, IGF-1R, IRS-1, PTEN, Akt, GSK3α, and GSK3β) and mTOR (TSC2, mTOR, P70S6K, and RPS6) pathways in 11 diverse tissues [liver, kidney, lung, aorta, two brain regions (cortex and cerebellum), and two predominantly slow-twitch and three predominantly fast-twitch skeletal muscles] from AL and CR rats, we have tested the widely held idea that CR induces the down-regulation of the insulin/IGF-1 and/or mTOR pathways. AL and CR rats were studied under two conditions: with endogenous insulin levels (i.e., AL>CR) and with insulin infused during a hyperinsulinemic-euglycemic clamp so that plasma insulin concentrations were closely matched between the two diet groups. We hypothesized that CR would induce tissue-specific effects on selected aspects of the insulin/IGF-1 and mTOR signaling pathways and that CR's effects on both pathways would be modulated by insulin infusion.

## Methods

### Materials

Reagents and apparatus for SDS-PAGE and immunoblotting were from Bio-Rad Laboratories (Hercules, CA). MILLIPLEX_MAP_ Cell Signaling Buffer and Detection Kit (#48-602), MILLIPLEX MAP 11-Plex Akt/mTOR Phosphoprotein Panel (#48-611; Akt^Ser473^, GSK3α^Ser21^, GSKβ^Ser9^, IGF1R^Tyr1135/1136^, IR^Tyr1162/1163^, IRS1^Ser312^, mTOR^Ser2448^, p70 S6 kinase^Thr412^, PTEN^Ser380^, RPS6^Ser235/236^, and TSC2^Ser939^), and total MAPmates for Akt (#46-605), GSK3β (#46-689), IGF1R (#46-646), IR (#46-687), IRS1 (#46-628), mTOR (#46-685), p70 S6 kinase (#46-630), and PTEN (#46-678) were all purchased from Millipore (Billerica, MA). Anti-GSK3α (#9338), anti-RPS6 (#2217), anti-TSC2 (3612), and anti-rabbit IgG-horseradish peroxide conjugate (#7074) were purchased from Cell Signaling Technology (Danvers, MA). Unless otherwise noted, all other reagents were purchased from Sigma Chemical (St. Louis, MO) or Fisher Scientific (Hanover Park, IL).

### Animal Treatment

Procedures for animal care were approved by the University of Michigan Committee on Use and Care of Animals. Male Fischer 344 x Brown Norway, F1 generation rats (FBN) were obtained at 3 months of age from Harlan (Indianapolis, IN). This strain of rats has previously been demonstrated to respond to CR with increased lifespan [21]. Rats were housed individually in shoebox cages and maintained on a 12∶12 h light-dark cycle (lights out at 17:00 h). Animals had free access to food chow (Lab Diet; PMI Nutritional International, Brentwood, MO) and water for a 2 wk acclimation period. Animals then had free access to food (NIH31 chow, TestDiet, Richmond, IN) and water for another 2 wk acclimation period. During this time, we measured food consumption of all rats between 15:30 and 16:30 h every day to determine baseline food intake (difference between food provided and food remaining). After the acclimation period, rats were ranked by weight (lowest to highest) and alternately assigned to the *ad libitum* control group (AL) and the calorie restriction treatment group (CR) so that the initial mean weight and standard error were similar for both groups. After the 2 wk acclimation, the AL group received *ad libitum* access to the NIH31 chow for the duration of the study. The CR group received NIH31/NIA fortified chow (TestDiet). The CR group was restricted to 60% of AL intake gradually over 3 weeks (90%, 75%, 60%). After this time, the CR group received 60–65% of AL intake daily for approximately 6 mo (between 182 and 200 days). All CR rats were fed between 15:30 and 16:30 h each day and food intake of both groups was measured daily. CR intake was adjusted weekly to 60–65% of AL intake. All rats were weighed weekly at the same time of day.

### Placement of Catheters

After approximately 6 mo of the feeding protocol, rats from both diet groups had catheters surgically placed into the jugular vein and carotid artery to sample blood. All surgical instruments were initially autoclaved and sterilized using a hot glass bead sterilizer. Rats were anesthetized with an intraperitoneal injection of ketamine (50–90 mg·kg^−1^) and xylazine (5–10 mg kg^−1^). The ventral neck and back of the head was shaved and the skin was prepared with 3 alternating scrubs of iodine and 70% ethanol. Under aseptic conditions, a small incision was made superior to the clavicle, exposing the carotid artery and jugular vein. Both vessels were catheterized and ligated in place with non-absorbable suture. Catheters were tunneled subcutaneously using a 16-gauge needle and exteriorized at the back of the neck via stainless steel connectors that were coated with medical silicon and fixed subcutaneously upon the closure of the incision. The catheters were filled with heparinized saline and tightly plugged with stainless steel surgical wire. Post-operatively, animals were placed in an isolator equipped with a heating pad for recovery. Ampicillin (100 mg·kg^−1^, intravenously) was given and if needed, buprenorphine (0.01–0.5 mg kg^−1^, subcutaneously) for pain every 8–12 h. Warm ringers solution was given to replace lost fluids (1 ml/100 g bw^−1^ h^−1^ surgery).

### Euglycemic-hyperinsulinemic Clamp (clamp) and Saline Infusion

A week after the catheterization surgery rats underwent the terminal experiment. At approximately 08:00 on the morning of clamps and saline infusion experiments, food was removed from rats (∼5 h prior to the start of the clamp and infusion procedures). The clamp protocol consisted of a 120 min experimental clamp period (t = 0 to 120 min). At t = −10 min, a blood sample (∼100 µl) was taken from the arterial catheter for assessment of basal levels of insulin and glucose. The insulin infusion clamp was begun at t = 0 with a primed-continuous infusion of human insulin (Novo Nordisk, Princeton, NJ). In addition, saline-infused rats were used to evaluate insulin signaling and glucose uptake in rats under basal conditions (without exogenous insulin infusion). Blood glucose was maintained at ∼120 mg·dL^−1^ during the clamp by measuring blood glucose with a glucometer (Accu-Check, Roche, Indianapolis, IN) every 10 min starting at t = 0 and infusing 50% dextrose (Hospira, Lake Forest, IL) at variable rates accordingly. The glucose infusion rate (GIR) was determined from the average of the infusion rates over the duration of the clamp protocol. Plasma insulin concentrations were determined from samples taken at t = −10 and 120 min from the venous catheter using a commercially available ELISA kit (#EZRNI-13K, Millipore). Our group has previously demonstrated that CR versus AL rats have an elevated C-peptide-to-insulin ratio, which is consistent with a CR-related elevation in insulin clearance [22]. Also consistent with this interpretation, Feuers and colleagues demonstrated that CR rats have compared to AL rats injected with radiolabeled insulin have greater insulin binding in the liver [23]. Accordingly, to achieve the goal of similar plasma values for insulin in both diet groups, the insulin infusion rate was lower for AL rats (4.0 mU·kg^−1^·min^−1^) compared to CR rats (4.7 to 6.0 mU·kg^−1^·min^−1^). At the end of 120 min, the rats were rapidly anesthetized by injecting pentobarbital into the venous catheter, and upon loss of pedal reflexes, five skeletal muscles [soleus (SOL), epitrochlearis (EPI), plantaris (PLN), tibialis anterior (TA), and adductor longus (ADL)] and six non-skeletal muscle tissues [kidney (KID), liver (LVR), cerebellum (CBM), brain cortex (CTX), lung (LNG), and the descending aorta (AOR)] were excised from each rat.

### Tissue Lysate Preparation

Frozen tissues were weighed, and transferred to pre-chilled glass grinding tubes (Kontes, Vineland, NJ), and homogenized in ice-cold lysis buffer (1 ml/muscle) using a glass pestle attached to a motorized homogenizer (Caframo, Wiarton, ON). The lysis buffer contained T-PER Tissue Protein Extraction Reagent (#78510, Fisher Scientific), 1 mM EDTA, 1 mM EGTA, 2.5 mM sodium pyrophosphate, 1 mM sodium vanadate, 1 mM ß-glycerophosphate, 1 µg/ml leupeptin, and 1 mM phenylmethylsulfonyl fluoride. Homogenates were transferred to microfuge tubes, rotated for 1 h at 4°C, and then centrifuged at 15,000 *g* for 15 min at 4°C to remove insoluble material. Protein concentration was measured using the bicinchoninic acid method (#23225, Fisher Scientific).

### Multiplex Analysis

An aliquot of lysate from each tissue was used to determine the phosphorylation status of 11 proteins in the Akt/mTOR signaling axis (Akt^Ser473^, GSK3α^Ser21^, GSKβ^Ser9^, IGF1R^Tyr1135/1136^, IR^Tyr1162/1163^, IRS1^Ser312^, mTOR^Ser2448^, p70 S6 kinase^Thr412^, PTEN^Ser380^, RPS6^Ser235/236^, and TSC2^Ser939^) using a commercially available kit (#48-611, Millipore). Another aliquot from each tissue sample was used in a second multiplex assay to determine the relative abundance of 8 proteins (Akt, GSK3β, IGF1R, IR, IRS1, mTOR, p70 S6 kinase, and PTEN). The relative abundance of the remaining 3 proteins (GSK3α, TSC2, and RPS6) was determined by immunoblotting as described below because antibody-bead conjugates for these proteins were not available for multiplex analysis at the time of the analysis. Multiplex analysis was performed by the Luminex L200 instrument (Luminex, Austin, TX), and data was analyzed by xPONENT software (Luminex).

### Immunoblotting

Equal amounts of protein from each tissue lysate were mixed with 6× Laemmli buffer, boiled with SDS loading buffer for 5 min, separated by 10% SDS-PAGE, and then transferred to nitrocellulose. Membranes were rinsed with Tris-buffered saline plus Tween (TBST; 0.14 M NaCl, 0.02 M Tris base, pH 7.6, and 0.1% Tween), blocked with 5% bovine serum albumin (BSA) in TBST for 1 h at room temperature and transferred to primary antibody 1∶1000 (either GSK3α, RPS6, or TSC2) in TBST plus 5% BSA overnight at 4°C. Blots were washed 3×5 min with TBST and incubated in buffer containing rabbit secondary antibody (1∶20,000 dilution) for 1 h at room temperature. Membranes were then washed 3×5 min with TBST and subjected to enhanced chemiluminescence (#34075, Fisher Scientific) for visualization of protein bands. Immunoreactive proteins were quantified by densitometry (AlphaEase FC, Alpha Innotech, San Leandro, CA).

### Statistical Analyses

Data were analyzed using SigmaPlot, version 11.0 (Systat Software Inc., San Jose, CA). Student's t-test was used for comparisons between the two diet groups (AL vs. CR) for body mass and GIR. Two-way ANOVA was used to determined significant main effects (diet, CR vs. AL; and infusate, saline vs. insulin) and interactions for plasma concentrations. Pearson product moment correlations were used to determine the relationship between signaling proteins. A *P* value≤0.05 was accepted as statistically significant for these aforementioned comparisons. Protein phosphorylation data were expressed as a ratio of phosphorylated to total protein abundance. Two-way ANOVA was used to determined significant main effects for protein signaling data. To take into account multiple hypotheses tested and to reduce the probability of reporting false positives, we used a Bonferroni correction, whereby the generally accepted level of significance (i.e. P value≤0.05) was divided by 363 (11 analytes×11 tissues×3 comparisons, i.e., 2 main effects and 1 interaction). With this correction implemented, a P value≤0.000138 was accepted as statistically significant for the protein signaling data. All data are presented as mean ± SEM.

## Results

### Body Mass

As expected, AL vs. CR rats had a significantly (P≤0.05) larger body mass (saline-infused: 430.4±15.3 g vs. 261.0±4.7 g; and clamped: 420.8±10.5 g vs. 272.7±9.7 g).

### Blood Glucose, GIR, and Plasma Insulin Levels

Prior to the clamp, there was a main effect of diet (P≤0.05) for plasma glucose levels (CR<AL) as there was a 10% decrease in the CR-INS group compared to the AL-INS group, and a 6% decrease in the CR-SLN group compared to the AL-SLN group (Table 1). During the infusion period, blood glucose was significantly greater (P≤0.05) for insulin infused vs. saline infused rats. As planned, blood glucose was successfully maintained at similar levels between the AL-INS and CR-INS group (119.6 mg·dL^−1^ vs. 121.5 mg·dL^−1^, respectively; Table 1). The GIR was 109% greater (P≤0.05) in the CR-INS group (34.3±2.2 mg·kg^−1^·min^−1^) compared to the AL-INS group (16.4±0.7 mg·kg^−1^·min^−1^).

**Table 1:** Blood glucose and plasma insulin concentrations.

	AL-SLN	CR-SLN	AL-INS	CR-INS	ANOVA
Blood Glucose Pre-Infusion (mg·dL^−1^)	112.3±2.0	106±2.3	112.7±2.5	101.5±4.0	*Diet
Blood Glucose Avg. during Infusion (mg·dL^−1^)	106.0±2.3	98.4±3.1	119.6±3.1	121.5±4.7	*Insulin
Plasma Insulin Pre-Infusion (µU·mL^−1^)	55.7±4.0	30.0±5.0	61.7±5.0	31.2±3.8	*Diet
Plasma Insulin Post Infusion (µU·mL^−1^)	43.9±6.7	25.2±3.5	164.6±11.1	159.9±9.8	*Insulin

Values are means ± SEM. n = 6 rats per treatment group.

* P≤0.05, Main Effect of Diet or Insulin Infusion.

Prior to the infusion period, there was a main effect of diet (P≤0.05) for plasma insulin levels (CR<AL) as the CR-SLN group had 46% lower plasma insulin levels compared to the AL-SLN group and the CR-INS group had 49% lower plasma insulin compared to the AL-INS group (Table 1). At the end of the clamp protocol, there was a main effect of insulin (P≤0.05) for plasma insulin levels (INS>SLN). As intended, the CR-INS (159.9 µU·mL^−1^) and AL-INS (164.6 µU·mL^−1^) groups were successfully elevated to very similar values (Table 1).

### Protein Phosphorylation

**Figure 1:**
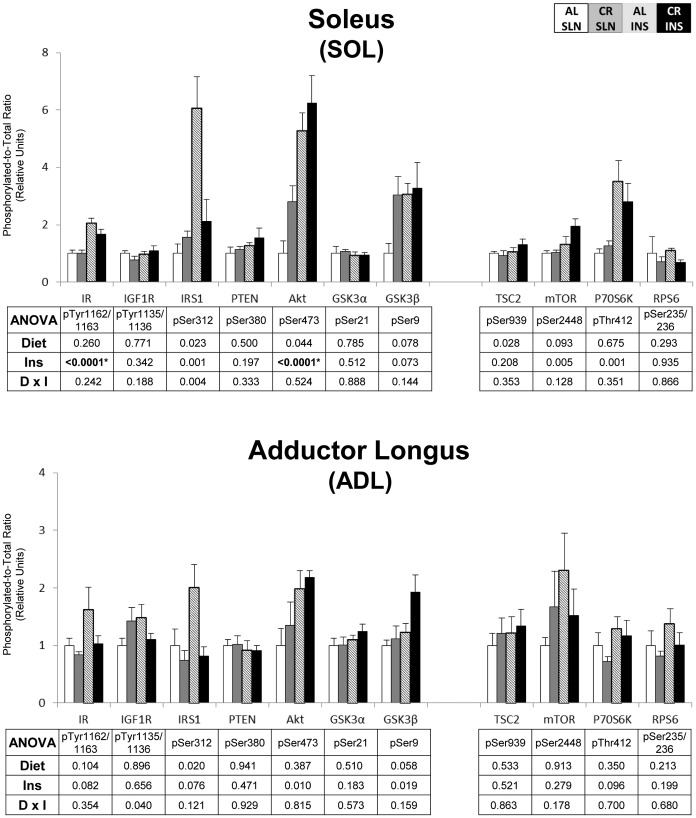
Insulin/IGF-1 and mTOR signaling in predominantly slow-twitch muscles. Open bars are the AL-SLN group, gray bars are the CR-SLN group, hatched bars are the AL-INS group, and black bars are the CR-INS group. Values are expressed as phosphorylated-to-total protein ratio. Main effects of Diet, Insulin Infusion (Ins), and Diet×Insulin Infusion Interactions (D×I) from 2-way ANOVA are shown in each panel. *P≤0.000138. Data are means ± SEM. n = 6 rats per diet group and treatment.

**Figure 2:**
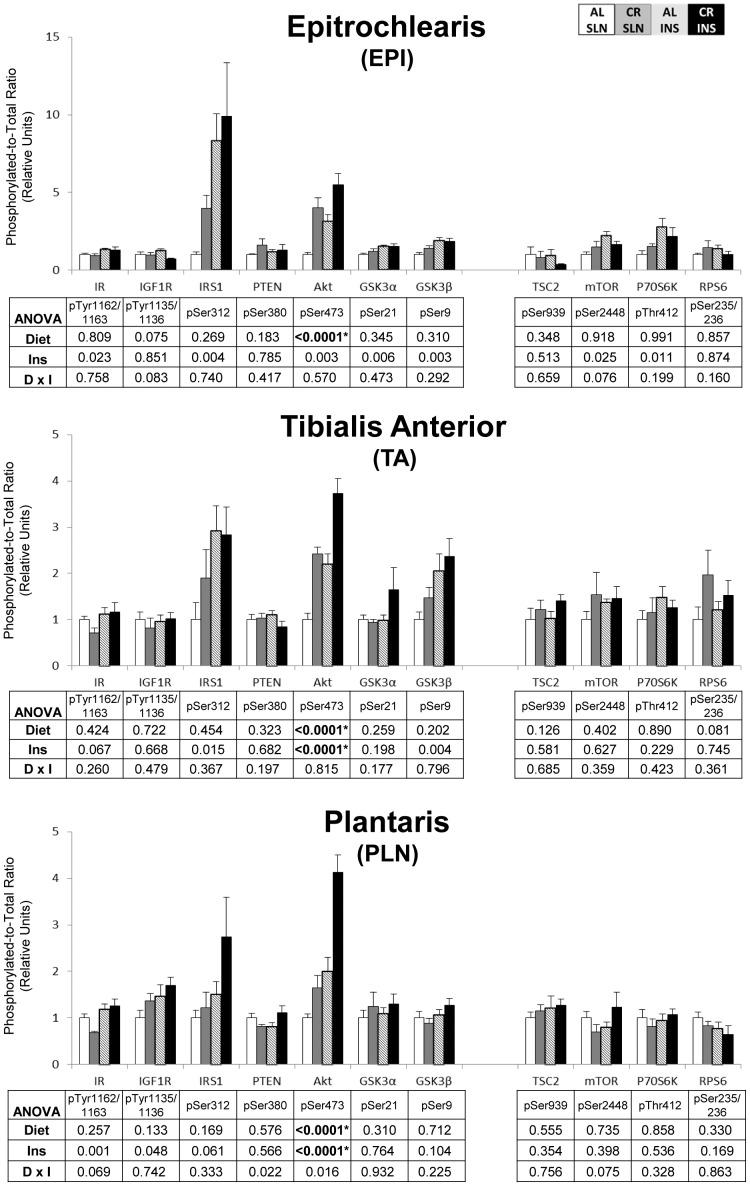
Insulin/IGF-1 and mTOR signaling in predominantly fast-twitch muscles. Open bars are the AL-SLN group, gray bars are the CR-SLN group, hatched bars are the AL-INS group, and black bars are the CR-INS group. Values are expressed as phosphorylated-to-total protein ratio. Main effects of Diet, Insulin Infusion (Ins), and Diet×Insulin Infusion Interactions (D×I) from 2-way ANOVA are shown in each panel. *P≤0.000138. Data are means ± SEM. n = 6 rats per diet group and treatment.

**Figure 3:**
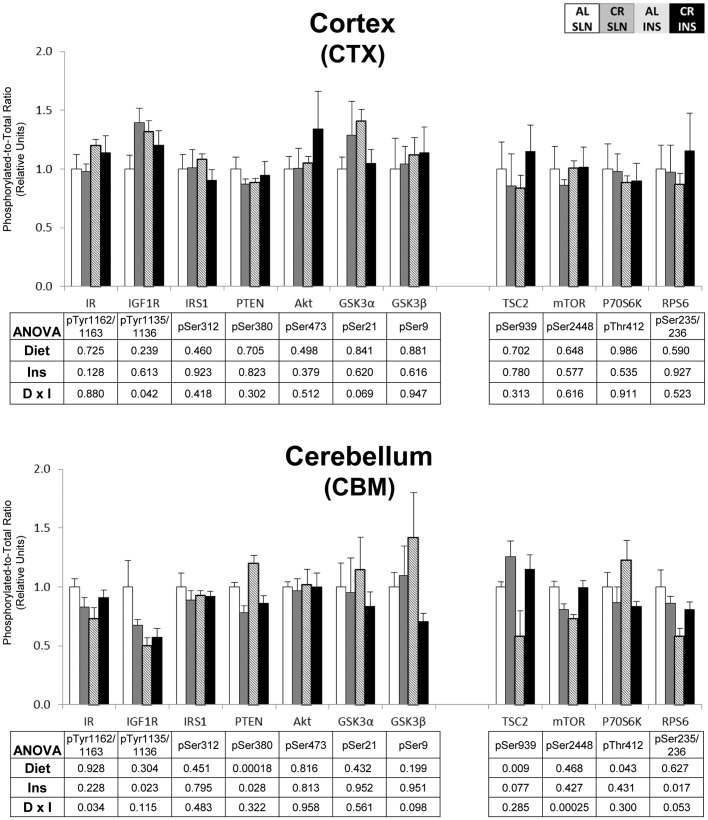
Insulin/IGF-1 and mTOR signaling in the brain. Open bars are the AL-SLN group, gray bars are the CR-SLN group, hatched bars are the AL-INS group, and black bars are the CR-INS group. Values are expressed as phosphorylated-to-total protein ratio. Main effects of Diet, Insulin Infusion (Ins), and Diet×Insulin Infusion Interactions (D×I) from 2-way ANOVA are shown in each panel. Data are means ± SEM. n = 6 rats per diet group and treatment.

**Figure 4:**
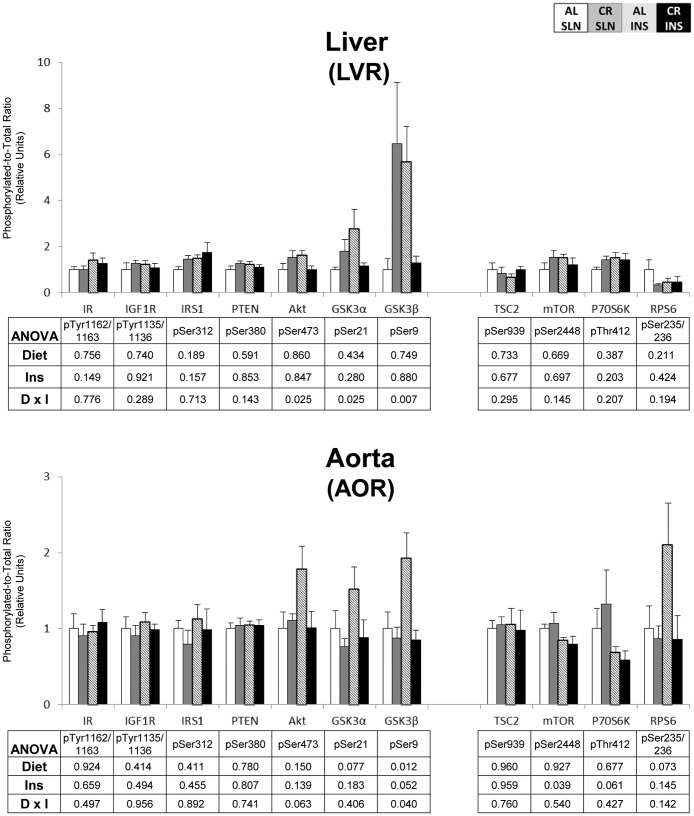
Insulin/IGF-1 and mTOR signaling in the liver and aorta. Open bars are the AL-SLN group, gray bars are the CR-SLN group, hatched bars are the AL-INS group, and black bars are the CR-INS group. Values are expressed as phosphorylated-to-total protein ratio. Main effects of Diet, Insulin Infusion (Ins), and Diet×Insulin Infusion Interactions (D×I) from 2-way ANOVA are shown in each panel. Data are means ± SEM. n = 6 rats per diet group and treatment.

**Figure 5:**
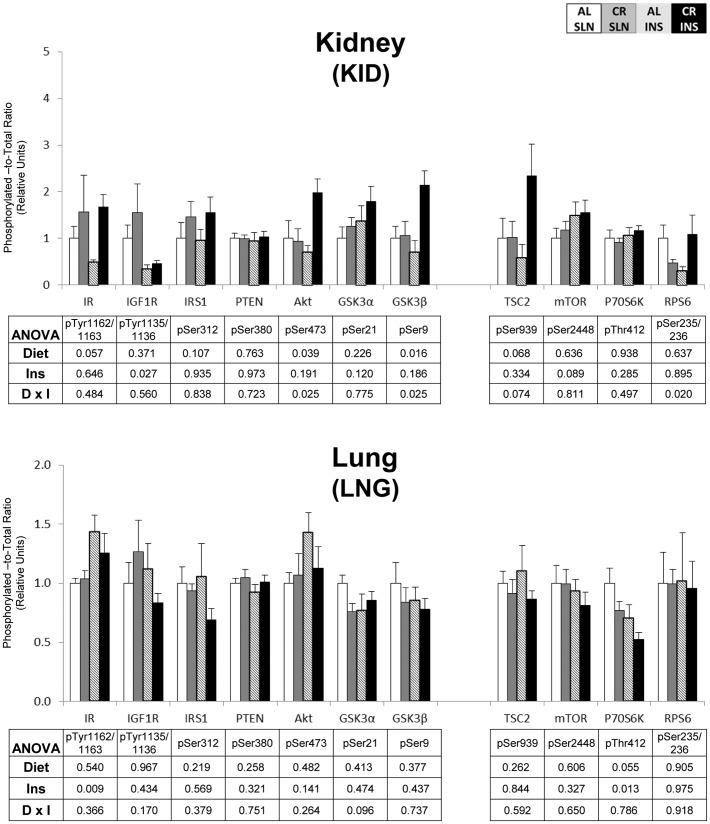
Insulin/IGF-1 and mTOR signaling in the kidney and lung. Open bars are the AL-SLN group, gray bars are the CR-SLN group, hatched bars are the AL-INS group, and black bars are the CR-INS group. Values are expressed as phosphorylated-to-total protein ratio. Main effects of Diet, Insulin Infusion (Ins), and Diet×Insulin Infusion Interactions (D×I) from 2-way ANOVA are shown in each panel. Data are means ± SEM. n = 6 rats per diet group and treatment.

To eliminate false positives due to multiple comparisons in the protein phosphorylation data, we used a high stringency statistical threshold (P≤0.000138). The most consistent effects were found for insulin signaling in skeletal muscle. For pAkt^Ser473^, there was a significant (P≤0.000138) main effect of diet (CR>AL) in the EPI, TA, and PLN, and a significant (P≤0.000138) main effect of insulin infusion (INS>SLN) in the SOL, TA, and PLN (Figs. 1 & 2). There was also a significant (P≤0.000138) main effect of insulin infusion (INS>SLN) in the SOL for pIR^Tyr1135/1136^ (Fig. 1). There were no significant effects of diet or insulin infusion in any of the other tissues (Figs. 3, 4, 5).

### Correlations

We calculated correlations between the phosphorylation of Akt and p70S6K and the phosphorylation of the respective substrates of these two kinases (Table 2). There was a significant (P≤0.05) positive correlation between pAkt^Ser473^ and pGSK3α^Ser21^ in the TA. In the non-skeletal muscle tissues, there was a significant (P≤0.05) positive correlation between pAkt^Ser473^ and pGSK3α^Ser21^ in the LVR, AOR, and KID. In 4 of the 5 muscles studied there was a significant (P≤0.05) positive correlation between pAkt^Ser473^ and pGSK3β^Ser9^). In all 6 of the non-skeletal muscles tissues, there was significant (P≤0.05) relationship between pAkt^Ser473^ and pGSK3β^Ser9^. There was a significant (P≤0.05) positive correlation between pAkt^Ser473^ and pTSC^Ser939^ in both of the predominantly slow-twitch muscles (SOL and ADL), but not in any of the predominantly fast-twitch muscles (EPI, TA and PLN). In the non-skeletal muscles tissues, there was significant (P≤0.05) positive correlation between pAkt^Ser473^ and pTSC2^Ser939^ in the CTX, KID, and LNG. There was a significant (P≤0.05) positive correlation between pP70S6K^Thr412^ and pRPS6^Ser235/236^ in 4 of the 5 muscles. In the non-skeletal muscle tissues, there was a significant (P≤0.05) positive correlation between pP70S6K^Thr412^ and pRPS6^Ser235/236^ in the CTX and KID.

**Table 2:** Correlations.

	pAkt^Ser473^ vs. pGSK3α^Ser21^	pAkt^Ser473^ vs. pGSK3β^Ser9^	pAkt^Ser473^ vs. pTSC2^Ser939^	pP70S6K^Thr412^ vs. pRPS6^S235/S236^
	R Value	R Value	R Value	R Value
**SOL**	0.043	0.564*	0.463*	0.57*
**ADL**	0.420	0.487*	0.691*	0.690*
**EPI**	0.386	0.625*	−0.248	0.108
**TA**	0.502*	0.690*	0.293	0.482*
**PLN**	0.114	0.350	0.107	0.531*
**CTX**	0.003	0.779*	0.753*	0.597*
**CBM**	0.336	0.545*	−0.037	0.146
**LVR**	0.681*	0.923*	−0.357	−0.094
**AOR**	0.796*	0.889*	0.138	−0.115
**KID**	0.479*	0.887*	0.747*	0.463*
**LNG**	0.210	0.405*	0.604*	0.093

* P≤0.05.

### Protein Abundance

There were no significant effects of diet or insulin infusion on protein abundance in the 11 tissues studied (Figures S1, S2, S3, S4, S5).

## Discussion

Calorie restriction (CR) has been demonstrated to extend normal lifespan and improve health in many species. There is a great deal of speculation that many of the beneficial effects of CR are attained, at least in part, by suppression of the insulin/IGF-1 and/or TOR signaling pathways. Remarkably, only a few studies have assessed the frequent assertion that CR causes attenuated activation of these pathways. Accordingly, we performed the current study as the initial step in probing the effects of CR and insulin on insulin/IGF-1 and mTOR signaling of multiple tissues in vivo. We determined protein phosphorylation of several key signaling proteins comparing AL and CR rats in two different conditions: 1) endogenous insulin levels (∼2-fold greater insulin for AL vs. CR), and 2) with insulin elevated to a similar level in both diet groups. Consistent with our hypothesis, we found that CR induced tissue-specific effects on certain aspects of the insulin/IGF-1 signaling pathway. Furthermore, the results do not support the notion that CR leads to a general reduction of insulin signaling. Rather, we observed an increase in pAkt in several skeletal muscles for CR compared to AL rats, and no evidence for a uniform change in insulin signaling of the other tissues of CR rats. Additionally there were no CR-related effects on the mTOR pathway proteins, and this pathway was also not consistently attenuated by CR in any of the tissues studied.

It is useful to interpret the current results for the insulin signaling pathway in skeletal muscle that were sampled after in vivo insulin infusion together with the results from a series of our previous experiments investigating the effects of CR on insulin signaling of isolated skeletal muscles [24], [25], [26], [27], [28], [29], [30], [31]. The most consistent finding from the earlier studies with isolated muscles is that CR, compared to AL-fed rodents, are characterized by a robust increase in insulin-stimulated Akt phosphorylation [24], [26], [27]. In a recent study from our group [24], male 9-mo old FBN rats underwent the identical feeding protocol as the rats used in current study, had EPI (predominantly fast-twitch) and SOL (predominantly slow-twitch) muscles excised and analyzed for the effects of CR and insulin on signaling ex vivo. There was no significant difference in insulin receptor tyrosine phosphorylation (pIR) between AL and CR muscles (EPI or SOL) that were incubated either in the absence of insulin or with a physiologic insulin dose (200 µU·ml^−1^; similar to the resulting insulin concentration of the insulin-infused rats in the current study). There was also no difference between CR vs. AL rats for pAkt in either EPI or SOL muscles incubated without insulin. However, with a physiologic (200 µU/ml) insulin dose, pAkt was significantly greater in CR vs. AL rats in both muscles. The CR-related differences found in isolated muscles are not attributable to the direct influence of systemic factors (e.g., CR-related differences in blood flow, hormone concentrations, etc.). Therefore, in skeletal muscles from AL compared to CR rats studied at an equal physiologic insulin concentration ex vivo, activation of key aspects of the insulin signaling pathway are either similar or greater for CR to AL rats.

The current data extend these earlier studies for CR effects on insulin signaling in the EPI and SOL by assessing the SOL and EPI along with 3 other skeletal muscles (including the predominantly slow-twitch ADL and predominantly fast-twitch TA and PLN) under in vivo conditions. There was a significant Diet-induced effect on pAkt (CR>AL) in 3 of the 5 skeletal muscles that were studied (EPI, TA and PLN) with no evidence for a CR-associated decrement in pAkt in the other muscles studied (SOL and ADL). In none of the muscles was the greater pAkt attributable to greater pIR in CR vs. AL rats. The ∼2-fold greater GIR during the clamp for CR vs. AL rats with plasma insulin levels of ∼160 µU/ml insulin for both groups in the current study was very similar to the ∼2-fold greater GIR for CR (70% of AL intake for 4 years) vs. AL cynomologus monkeys undergoing a clamp with plasma insulin levels of ∼250 µU/ml in both diet groups [32]. Insulin-stimulated pAkt in the vastus lateralis was also greater for CR vs. AL monkeys. However, in contrast to the current results, pIR values were greater in the vastus lateralis of CR vs. AL monkeys. Previous research has also compared insulin signaling in hindlimb skeletal muscle from AL and CR mice after a large bolus of insulin (10 IU/kg) was injected into their portal vein [33], [34]. Consistent with results for either rats or monkeys, the pAkt from insulin-stimulated muscle was greater for CR vs. AL mice. There was also a CR-related increase in pIR in these mice. Differences among studies for CR effects on pIR may be related to differences between species and/or differences in the insulin-stimulation protocols (e.g., insulin dose, duration of exposure, etc.). We previously found significantly greater pIR for CR vs. AL rats in SOL and EPI muscles studied ex vivo with a supraphysiologic insulin dose (5000 µU/ml), but not with a physiologic insulin dose (200 µU/ml) [24]. Regardless, there was no evidence from any of these species that CR results in attenuated insulin signaling in skeletal muscle. Importantly, in the current study, even with endogenous insulin levels that were ∼50% lower than AL values, phosphorylation of IR and Akt was similar or greater for saline-infused CR rats in all 5 muscles.

A very limited number of previous studies have addressed the effects of CR on the insulin signaling pathway in non-skeletal muscle tissues. In the current study, the skeletal muscles were relatively consistent with regard to greater pAkt in response to insulin infusion and diet (CR>AL), but these changes in pAkt were absent for all other tissues that were studied. It is also notable that pAkt did not correlate with both of the two isoforms of pGSK3 (α and β) in every tissue. A positive correlation between pAkt and pGSK3β was consistently found in the 11 tissues (observed in 4 of 5 muscles with a trend in the PLN, and significant in all 6 non-muscle tissues), but significant correlations between pAkt and pGSK3α were only found in the TA, LVR, AOR, and KID. The differential responses of pGSK isoforms to Diet and Insulin infusion are not unexpected, as differences between the α and β isoforms have often been previously noted in other models encompassing a variety of cell and tissue types [35], [36], [37], [38], [39], [40]. Taken together, the results of the current study demonstrate that the effects of CR on the insulin signaling pathway, and specifically on pAkt, are not uniform, but are highly tissue-specific.

In recent years there has been a great deal of interest in the mTOR pathway as a potential target for regulating health and lifespan. Downregulation of the mTOR pathway through genetic or pharmacological manipulation has been reported to be beneficial for health and longevity in rodents [1], [9], [19]. Very few previous studies have investigated the effects of CR on mTOR signaling in rodents. Bonkowski et al. observed a decrease in pmTOR in mouse hindlimb muscle that was studied with endogenous insulin that was ∼40% lower for CR vs. AL mice [33]. Zheng et al. reported no CR-induced effect on pP70S6K values in the liver of rats with endogenous insulin values that were not reported [41]. In the current study, there was a significant correlation between pP70S6K and pRPS6 in 4 of the 5 muscles as well as the CTX and KID, but the data do not support the idea that marked attenuated mTOR signaling is a universal characteristic of CR. It is notable that the lack of CR-related attenuation of mTOR signaling was found using either a high stringency threshold for statistical significance with the Bonferroni correction or with a low stringency threshold in the absence of this adjustment.

In conclusion, CR has diverse, tissue-specific effects on the phosphorylation of key proteins in the insulin/IGF-1 signaling pathway. CR did not induce a uniform attenuation of insulin/IGF-1 signaling, rather the most striking and consistent effect of CR was elevated pAkt in skeletal muscle. There were no significant effects of CR on the phosphorylation of mTOR signaling pathway proteins, and there was no evidence for a general attenuation of the mTOR pathway. The current results provide the most comprehensive data set currently available for CR effects on the insulin/IGF1 and mTOR pathways in multiple tissues, and they do not provide evidence supporting the notion that the beneficial effects of CR may be largely attributable to a general downregulation of insulin/IGF-1 and/or mTOR signaling in many or most tissues. However, it is important to recognize that these results do not eliminate the possible roles of these pathways for specific effects of CR. For example, the enhanced pAkt in skeletal muscles is likely important for improved insulin-mediated glucose disposal with CR [24], [27]. Furthermore, CR effects on specific cell types may be obscured in whole tissue analyses. There may also be CR effects on both pathways that will be revealed by evaluating diurnal fluctuations in signaling events. Although the current results refute the idea that CR induces a uniform attenuation of insulin/IGF-1 and/or mTOR signaling in most tissues of 9 month-old rats, it will be important for future research to elucidate possible CR effects in older animals and in different species, as well as the more complex and nuanced influence of CR on these pathways in multiple tissues and cell types.

## Supporting Information

Figure S1 **Total protein abundance in predominantly slow-twitch muscles.** Open bars are the AL-SLN group, gray bars are the CR-SLN group, hatched bars are the AL-INS group, and black bars are the CR-INS group. Main effects of Diet, Insulin Infusion (Ins), and Diet×Insulin Infusion Interactions (D×I) from 2-way ANOVA are shown in each panel. Data are means ± SEM. n = 6 rats per diet group and treatment.
(TIF)

Figure S2 **Total protein abundance in predominantly fast-twitch muscles.** Open bars are the AL-SLN group, gray bars are the CR-SLN group, hatched bars are the AL-INS group, and black bars are the CR-INS group. Main effects of Diet, Insulin Infusion (Ins), and Diet×Insulin Infusion Interactions (D×I) from 2-way ANOVA are shown in each panel. Data are means ± SEM. n = 6 rats per diet group and treatment.
(TIF)

Figure S3 **Total protein abundance in the brain.** Open bars are the AL-SLN group, gray bars are the CR-SLN group, hatched bars are the AL-INS group, and black bars are the CR-INS group. Main effects of Diet, Insulin Infusion (Ins), and Diet×Insulin Infusion Interactions (D×I) from 2-way ANOVA are shown in each panel. Data are means ± SEM. n = 6 rats per diet group and treatment.
(TIF)

Figure S4 **Total protein abundance in the liver and aorta.** Open bars are the AL-SLN group, gray bars are the CR-SLN group, hatched bars are the AL-INS group, and black bars are the CR-INS group. Main effects of Diet, Insulin Infusion (Ins), and Diet×Insulin Infusion Interactions (D×I) from 2-way ANOVA are shown in each panel. Data are means ± SEM. n = 6 rats per diet group and treatment.
(TIF)

Figure S5 **Total protein abundance in the kidney and lung.** Open bars are the AL-SLN group, gray bars are the CR-SLN group, hatched bars are the AL-INS group, and black bars are the CR-INS group. Main effects of Diet, Insulin Infusion (Ins), and Diet×Insulin Infusion Interactions (D×I) from 2-way ANOVA are shown in each panel. Data are means ± SEM. n = 6 rats per diet group and treatment.
(TIF)
