# Supplementary figures and images for: Tissue-Specific Responses of IGF-1/Insulin and mTOR Signaling in Calorie Restricted Rats
Source: PLoS One. 2012 Jun 6;7(6):e38835. doi: 10.1371/journal.pone.0038835 (PMC3368930; doi:10.1371/journal.pone.0038835)

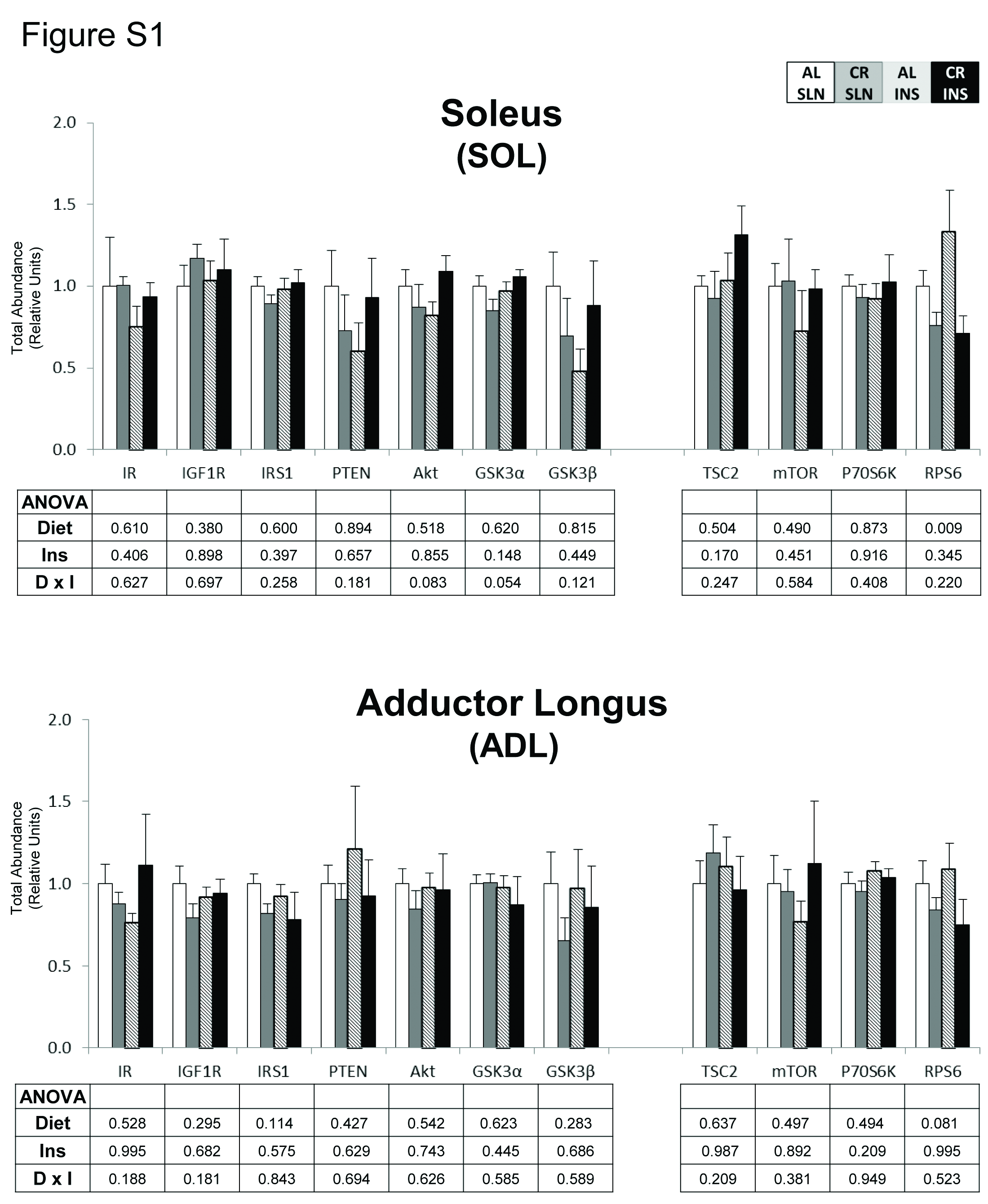

Supplement: Figure S1 — Total protein abundance in predominantly slow-twitch muscles. Open bars are the AL-SLN group, gray bars are the CR-SLN group, hatched bars are the AL-INS group, and black bars are the CR-INS group. Main effects of Diet, Insulin Infusion (Ins), and Diet×Insulin Infusion Interactions (D×I) from 2-way ANOVA are shown in each panel. Data are means ± SEM. n = 6 rats per diet group and treatment. (TIF) [file pone.0038835.s001.tif]

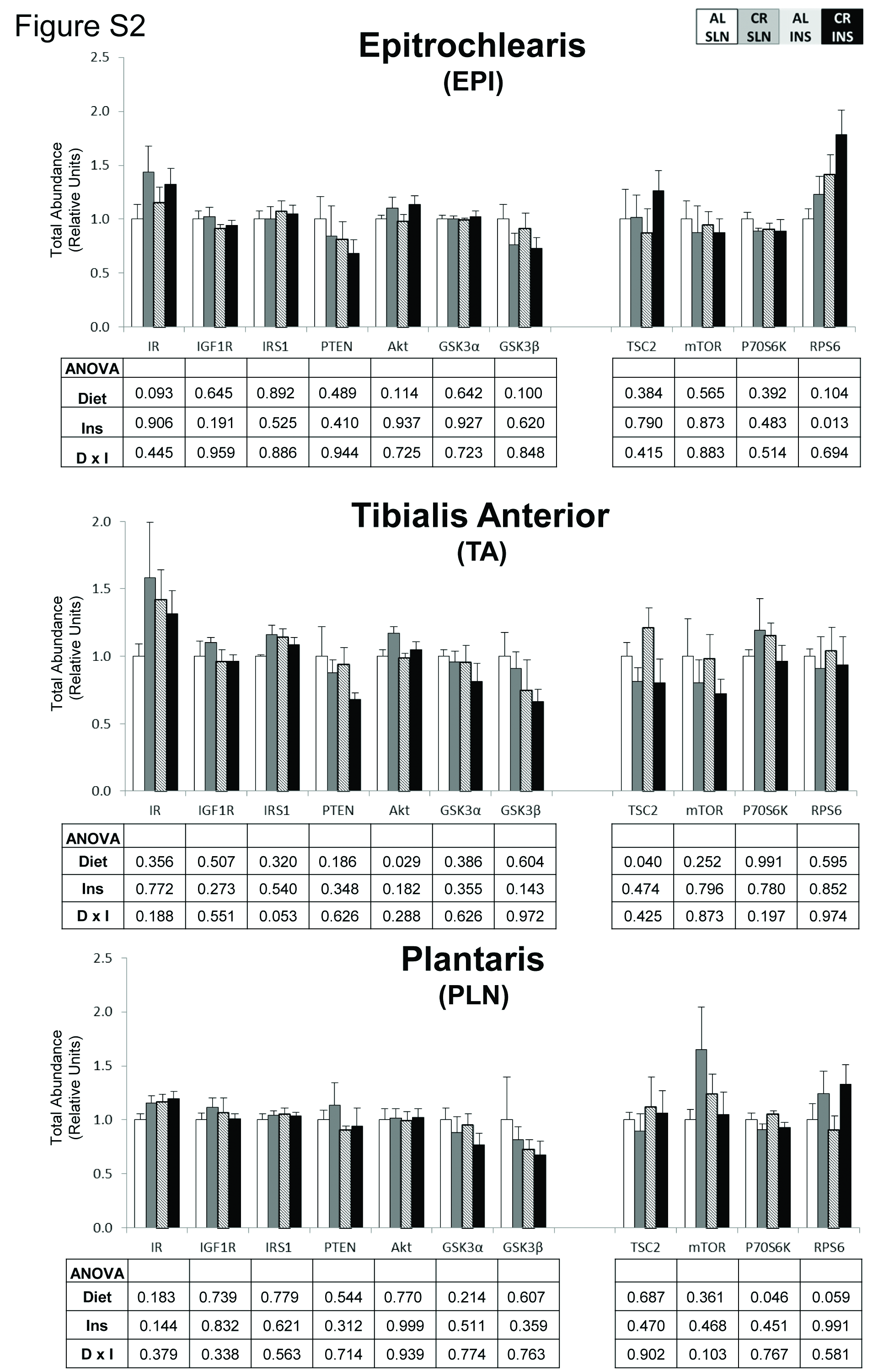

Supplement: Figure S2 — Total protein abundance in predominantly fast-twitch muscles. Open bars are the AL-SLN group, gray bars are the CR-SLN group, hatched bars are the AL-INS group, and black bars are the CR-INS group. Main effects of Diet, Insulin Infusion (Ins), and Diet×Insulin Infusion Interactions (D×I) from 2-way ANOVA are shown in each panel. Data are means ± SEM. n = 6 rats per diet group and treatment. (TIF) [file pone.0038835.s002.tif]

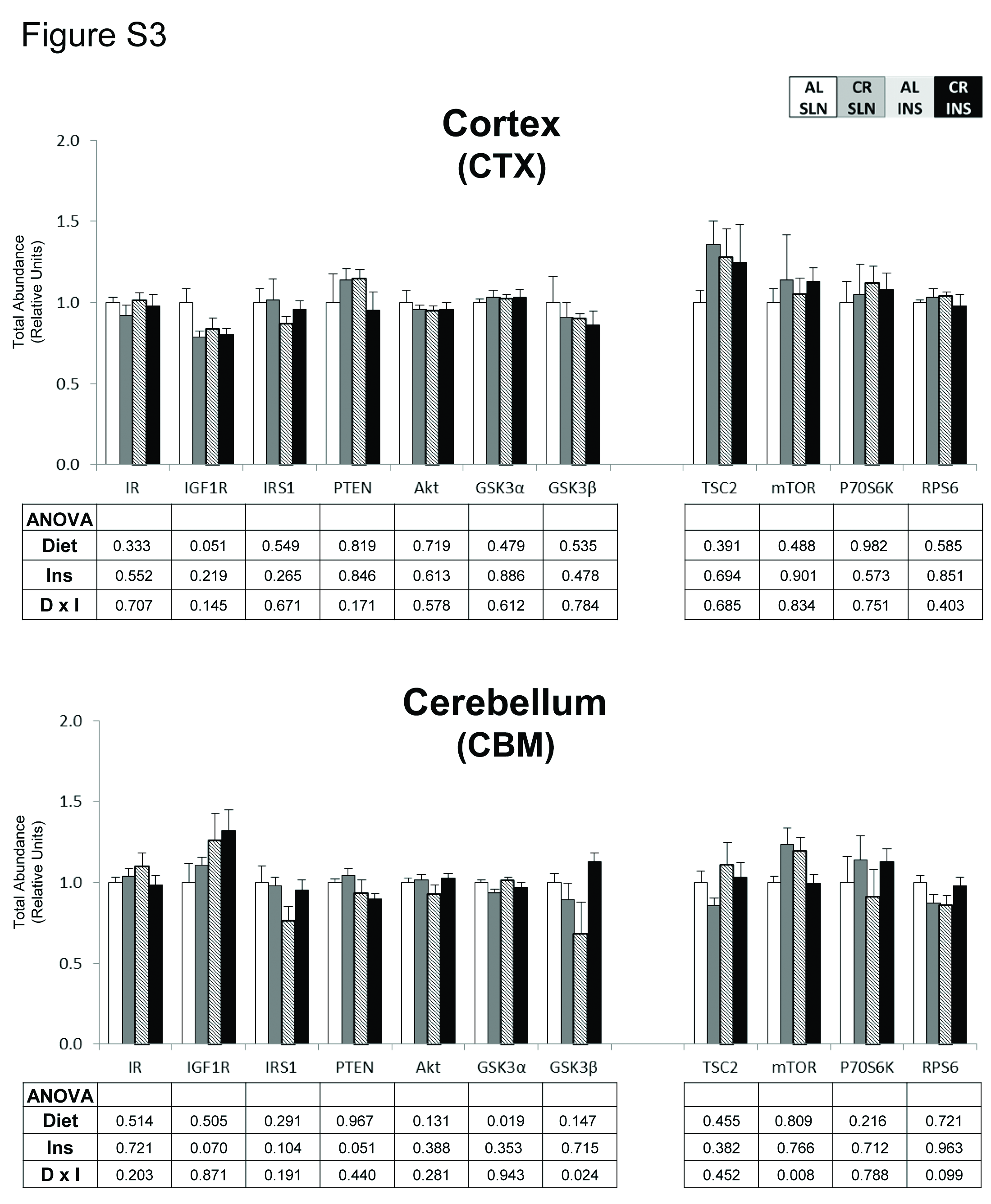

Supplement: Figure S3 — Total protein abundance in the brain. Open bars are the AL-SLN group, gray bars are the CR-SLN group, hatched bars are the AL-INS group, and black bars are the CR-INS group. Main effects of Diet, Insulin Infusion (Ins), and Diet×Insulin Infusion Interactions (D×I) from 2-way ANOVA are shown in each panel. Data are means ± SEM. n = 6 rats per diet group and treatment. (TIF) [file pone.0038835.s003.tif]

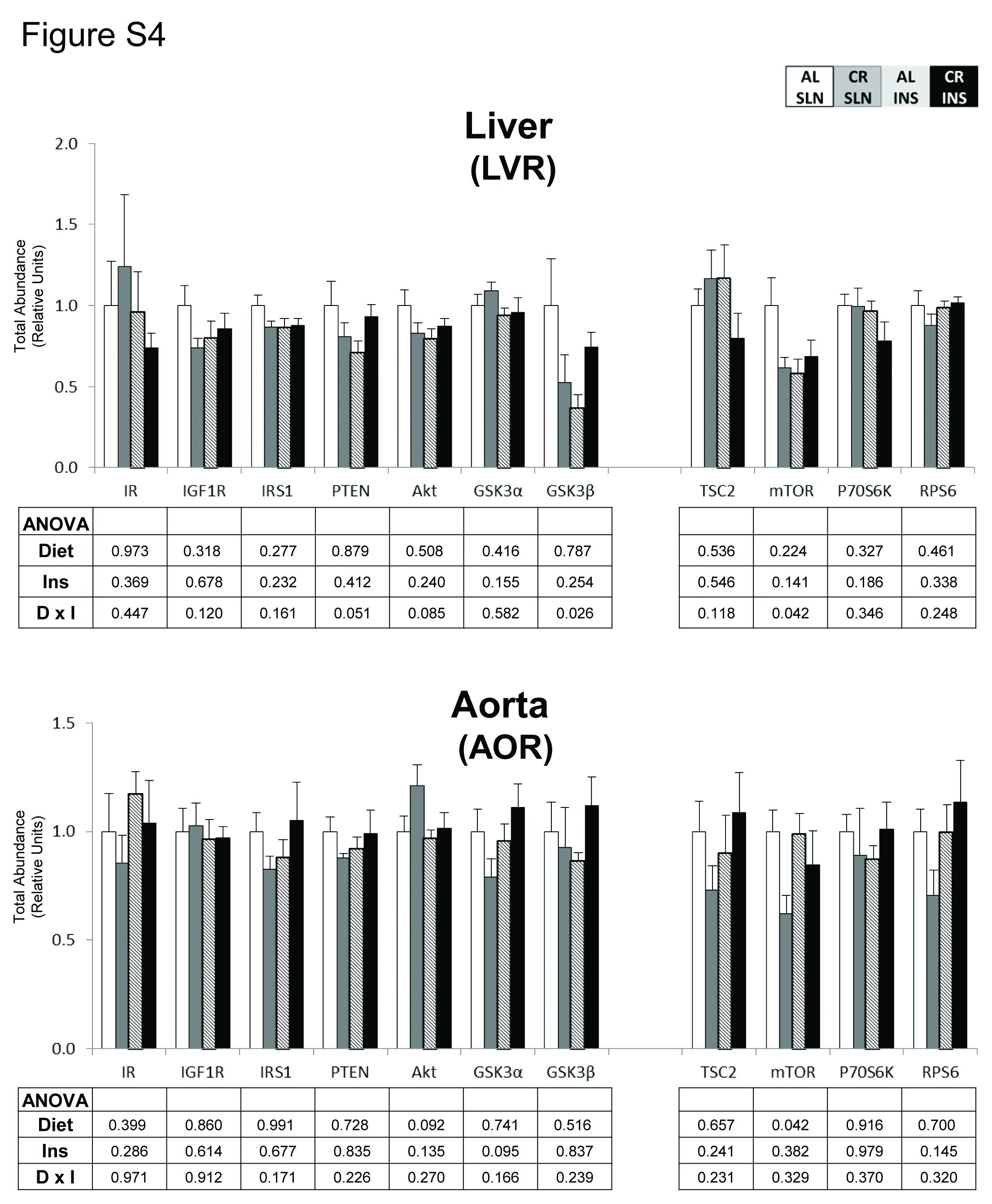

Supplement: Figure S4 — Total protein abundance in the liver and aorta. Open bars are the AL-SLN group, gray bars are the CR-SLN group, hatched bars are the AL-INS group, and black bars are the CR-INS group. Main effects of Diet, Insulin Infusion (Ins), and Diet×Insulin Infusion Interactions (D×I) from 2-way ANOVA are shown in each panel. Data are means ± SEM. n = 6 rats per diet group and treatment. (TIF) [file pone.0038835.s004.tif]

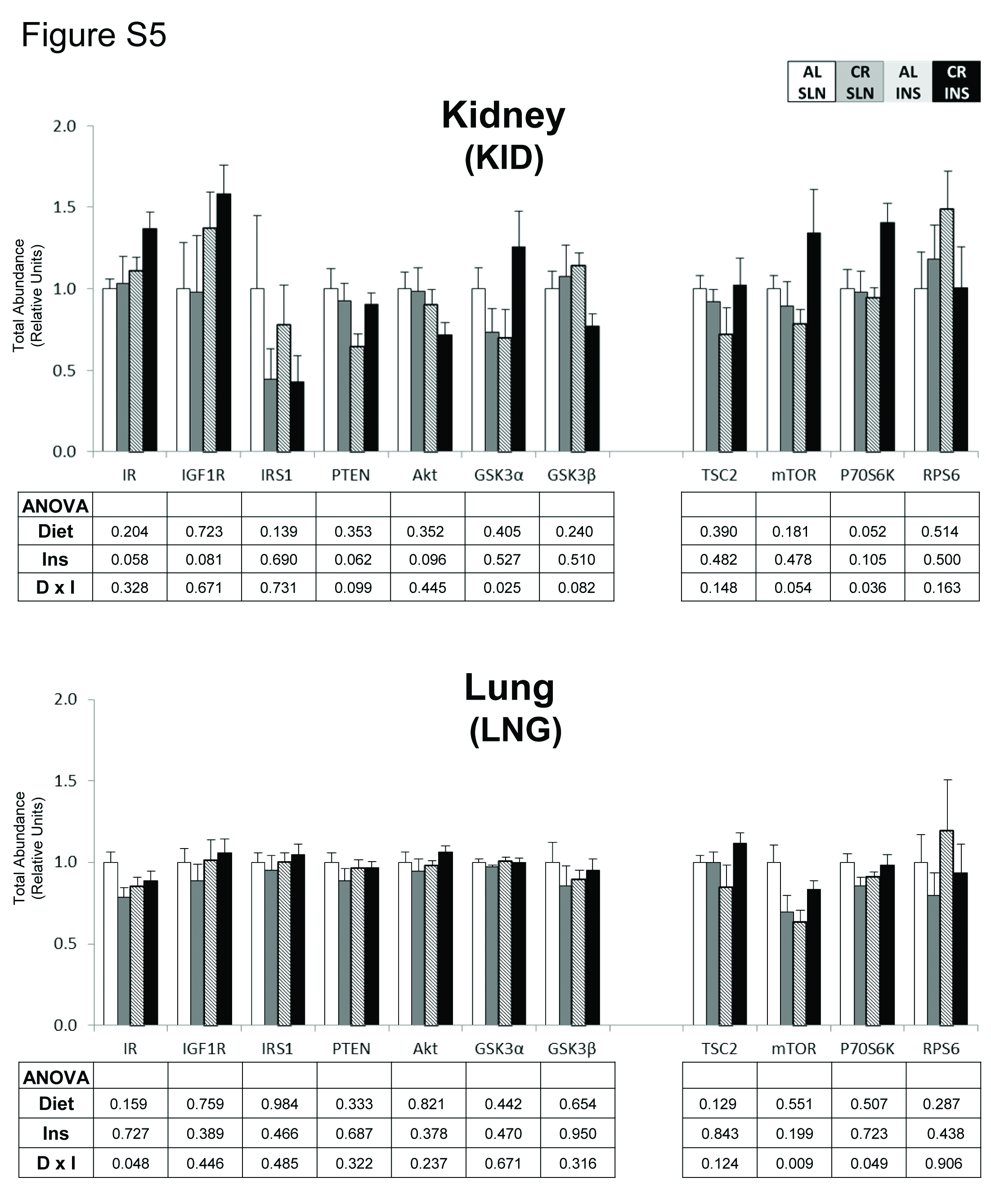

Supplement: Figure S5 — Total protein abundance in the kidney and lung. Open bars are the AL-SLN group, gray bars are the CR-SLN group, hatched bars are the AL-INS group, and black bars are the CR-INS group. Main effects of Diet, Insulin Infusion (Ins), and Diet×Insulin Infusion Interactions (D×I) from 2-way ANOVA are shown in each panel. Data are means ± SEM. n = 6 rats per diet group and treatment. (TIF) [file pone.0038835.s005.tif]
